# A time-calibrated molecular phylogeny of the precious corals: reconciling discrepancies in the taxonomic classification and insights into their evolutionary history

**DOI:** 10.1186/1471-2148-12-246

**Published:** 2012-12-18

**Authors:** Néstor E Ardila, Gonzalo Giribet, Juan A Sánchez

**Affiliations:** 1 Departamento de Ciencias Biológicas-Facultad de Ciencias, Laboratorio de Biología Molecular Marina (BIOMMAR), Universidad de los Andes, Carrera 1E No 18A-10, Bogotá, Colombia; 2 Department of Organismic and Evolutionary Biology and Museum of Comparative Zoology, Harvard University, Cambridge, MA, 02138, USA; 3 Present address: Departamento de Ciencias Básicas, Programa de Biología, Universidad de la Salle, Carrera 2 No. 10-70, Bogotá, Colombia

## Abstract

**Background:**

Seamount-associated faunas are often considered highly endemic but isolation and diversification processes leading to such endemism have been poorly documented at those depths. Likewise, species delimitation and phylogenetic studies in deep-sea organisms remain scarce, due to the difficulty in obtaining samples, and sometimes controversial. The phylogenetic relationships within the precious coral family Coralliidae remain largely unexplored and the monophyly of its two constituent genera, *Corallium* Cuvier and *Paracorallium* Bayer & Cairns, has not been resolved. As traditionally recognized, the diversity of colonial forms among the various species correlates with the diversity in shape of their supporting axis, but the phylogenetic significance of these characters remains to be tested. We thus used mitochondrial sequence data to evaluate the monophyly of *Corallium* and *Paracorallium* and the species boundaries for nearly all named taxa in the family. Species from across the coralliid range, including material from Antarctica, Hawaii, Japan, New Zealand, Taiwan, Tasmania, the eastern Pacific and the western Atlantic were examined.

**Results:**

The concatenated analysis of five mitochondrial regions (COI, 16S rRNA, ND2, and ND3-ND6) recovered two major coralliid clades. One clade is composed of two subgroups, the first including *Corallium rubrum*, the type species of the genus, together with a small group of *Paracorallium* species (*P. japonicum* and *P. tortuosum*) and *C. medea* (clade I-A); the other subgroup includes a poorly-resolved assemblage of six *Corallium* species (*C. abyssale, C. ducale, C. imperiale, C. laauense, C. niobe,* and *C. sulcatum*; clade I-B). The second major clade is well resolved and includes species of *Corallium* and *Paracorallium* (*C. elatius, C. kishinouyei, C. konojoi, C. niveum, C. secundum, Corallium* sp., *Paracorallium nix, Paracorallium thrinax* and *Paracorallium* spp.). A traditional taxonomic study of this clade delineated 11 morphospecies that were congruent with the general mixed Yule-coalescent (GMYC) model. A multilocus species-tree approach also identified the same two well-supported clades, being Clade I-B more recent in the species tree (18.0-15.9 mya) than in the gene tree (35.2-15.9 mya). In contrast, the diversification times for Clade II were more ancient in the species tree (136.4-41.7 mya) than in the gene tree (66.3-16.9 mya).

**Conclusions:**

Our results provide no support for the taxonomic status of the two currently recognized genera in the family Coralliidae. Given that *Paracorallium* species were all nested within *Corallium*, we recognize the coralliid genus *Corallium*, which includes the type species of the family, and thus consider *Paracorallium* a junior synonym of *Corallium*. We propose the use of the genus *Hemicorallium* Gray for clade I-B (species with long rod sclerites, cylindrical autozooids and smooth axis). Species delimitation in clade I-B remains unclear and the molecular resolution for Coralliidae species is inconsistent in the two main clades. Some species have wide distributions, recent diversification times and low mtDNA divergence whereas other species exhibit narrower allopatric distributions, older diversification times and greater levels of mtDNA resolution.

## Background

Delimiting species is an old systematic problem, which continues to be controversial (e.g., [1-9]). If species delimitation estimates are based solely on mtDNA, groups can be influenced by the timing of speciation and the migration rates or dispersal capabilities of the species [10]. In addition, it is crucial to better understand species boundaries in deep-water groups. Estimation of divergence times in deep-sea faunas is a promising approach to understand events that have influenced both the evolution of these neglected marine organisms and the changes in this poorly explored environment.

Species are the fundamental units in many studies on systematics, biogeography, epidemiology, and conservation biology (e.g., [9,11]). Species also constitute the unit for assessing biodiversity and therefore accurate identification of individuals is crucial in many areas of inquiry. In systematics and biogeographical analyses, species are frequently used as terminal taxa in phylogenetic analysis. However, relatively little effort has focused on the process of identifying and delimitating such species. In addition, there are no universal criteria by which species should be delineated and identified, and both, non-tree based (measures of gene flow) and tree-based approaches have been applied [4,6,7,12-15].

In octocorals, species identification has traditionally been based on external morphology (axis, branching pattern, calyx morphology, polyp arrangement, surface texture, coloration, etc.) and sclerite composition [16-20]. The taxonomy of precious corals is particularly enigmatic, because many species were described based on small deep-sea fragmentary samples, limiting our understanding of their real intra-colony and intraspecific variation [21]. In addition, some descriptions are imprecise and the whereabouts of the type material remains unknown.

Molecular species delimitation usually uses tree-based methods to assess monophyly [1] or more recently have applied genealogical-based coalescent methods (e.g., [2-5,9,11,22-24]). Tree-based methods reconstruct the evolutionary relationships among individuals and look for reciprocal monophyly, but are often dependent on the method/model of tree inference and it is not uncommon that single-gene trees differ from concatenated gene-based inferences. Thus, concatenation of markers and multilocus coalescent approaches have been favoured more recently (e.g., [23,25]). Species-tree inference and estimation of divergence times from a single locus (often a mitochondrial gene) and multi-individual data are possible in a species tree framework [26], although these analyses are usually carried out using multilocus datasets [27]. However, coalescent-based approaches can show signal for lineage divergence despite the lack of monophyly in gene-trees, which can be common in recently diverged species [3]. Time-calibrated phylogenies have also the advantage of using general speciation models, which examine species boundaries from sequences by identifying evolving lineages bridging the coalescent with speciation in the phylogeny [5].

The particular interest on Coralliidae phylogenetics responds also to the intricate evolution of its axial characters and the questionable validity of its two currently recognized genera, in addition to aspects of their biology and conservation, as most species are of commercial interest for the jewelry industry. The diversity of colonial forms among the various species of precious corals corresponds with various shapes of the supporting axis. Branching varies from sparse, in *Corallium abyssale* Bayer, to profuse, in *Paracorallium japonicum* Kishinouye. Terminal branchlets vary from stout and blunt to slender and delicate. The surface of the axis may be smooth or distinctly marked by narrow longitudinal grooves underlying the coenenchymal canals and some species include pits [28,29]. Coenenchymal sclerites are basically of the radiate type, slender rods and smooth or rough double clubs [30-32].

*Corallium* and *Paracorallium*, the two accepted Coralliidae genera, comprise 17 and 7 described species, respectively. Most have been documented from the Pacific Ocean and only two species are from the eastern North Atlantic [21,29,31]. Coralliidae is closely related to Paragorgiidae [33-35], whose members lack a supporting axis. The most complete molecular phylogenetic hypothesis for Octocorallia [36] shows that Scleraxonia, the suborder containing Coralliidae, is polyphyletic. To date, limited molecular systematic studies of Coralliidae [33,35] have provided evidence for major discrepancies between phylogeny and generic taxonomy. Additional studies of precious corals include genetic surveys, radial growth rates, dating, and recruitment estimates [37,38], but little is known about their phylogenetic relationships. Furthermore, all precious corals are currently threatened not only by the jewelry industry but also the trawling activity on seamounts and other deep-sea rocky habitats [39-42]. However, not all precious coral species have been included in the CITES list (the Convention on International Trade in Endangered Species of Wild Fauna and Flora) due to difficult identification and uncertainty about population status.

In this study, before understanding the phylogenetic relationships within Coralliidae, it was important to delineate species boundaries with particular interest in the precise membership of the type species of the family, *Corallium rubrum* Linnaeus, and to re-evaluate the validity of the genus *Paracorallium* Bayer & Cairns. Here, we provide an extended phylogeny of Coralliidae based on a large geographical and broad taxon sampling and using data from five mitochondrial regions. We use these data for a genealogical approach to assess the significance of clustering and lineage divergence and delimit species of precious corals.

## Results

### Concatenated analysis

**Figure 1:**
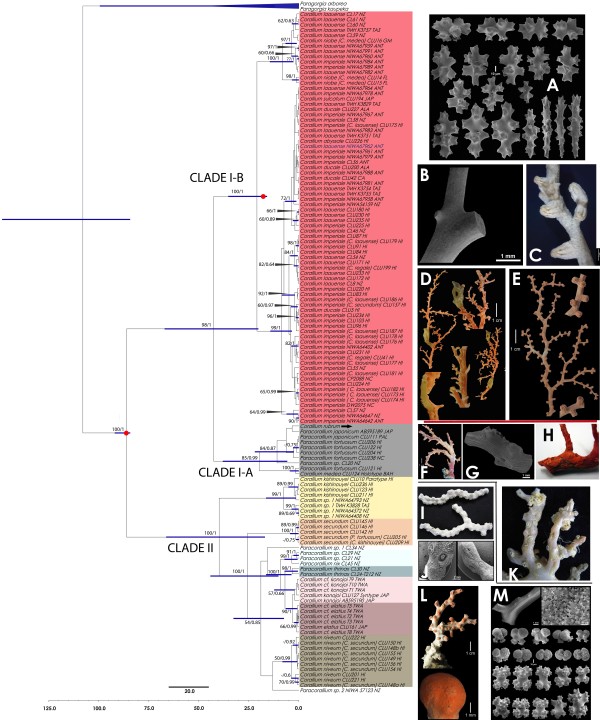
**Bayesian analysis of concatenated mtDNA.** Bayesian analysis of the concatenated dataset (COI+16S rRNA+16S-ND2+ ND3-ND6, 2263 bp, n = 129) performed with BEAST. Each node in the tree is labeled with its Maximum Likelihood boostrap (left) and Bayesian posterior probabilities (right) if it is greater than 50% or 0.5 respectively. The blue bars represent the 95% HPD interval for the divergence time estimates, which is only available for nodes with posterior probability <0.5. The red points represent the calibration node. The arrow indicates the position of the type species of the family, *Corallium rubrum*. Sampling localities are abbreviated as ALA: Alaska, ANT: Antarctica, BAH: Bahamas, CA: California, FL: Florida, GM: Gulf of Mexico, HI: Hawaii, JAP: Japan, NC: New Caledonia, NZ: New Zealand, PAL: Palau, TAS: Tasmania, TWA: Taiwan. Figures included, **A**. Sclerites of *C. laauense*, **B**. Axis of *C. laauense*, **C**. Autozooid polyps of *C. niobe*, **D**. Holotype colony of *C. imperiale* showing axis with typical tunnels and the commensal polychaets. **E**. Colony of *C. abyssale* showing typical cylindrical autozooids, **F**-**G** Colony and axis of *C. japonicum*, **H**. Colony of *C. rubrum*, **I**. Colony of *C. kishinouyei*, **J**. Colony of, **K**. Colony of *P. tortuosum*, **L**. Colony of *C. konojoi*, **M**. Axis and sclerites of *C. konojoi.* Misidentifications in the original material are presented in parentheses.

The phylogeny obtained from the concatenated approach for cytochrome *c* oxidase subunit I (COI), 16S ribosomal RNA (16S rRNA), NADH dehydrogenase subunit 2 (ND2), and NADH dehydrogenase subunits 3–6 (ND3-ND6) mtDNA, splits basally into two supported clades (Figure 1). Clade I is further divided into two subgroups, Clade I-A including *Corallium rubrum*, the type species of the genus, nested within a small group of *Paracorallium* species (*P. japonicum* and *P. tortuosum* Bayer) and *C. medea* Bayer. Clade I-B includes several *Corallium* species (*C. abyssale, C. ducale* Bayer*, C. imperiale* Bayer*, C. laauense* Bayer*, C. niobe* Bayer*,* and *C. sulcatum* Kishinouye) but several species sampled from multiple localities appear polyphyletic or paraphyletic. Such is the case of *C. imperiale* and *C. laauense,* which have a wide geographical distribution throughout the Pacific Ocean. *Corallium imperiale* has been reported from Hawaii, New Zealand, New Caledonia, Antarctica and California and *C. laauense* from Hawaii, New Zealand, Tasmania and Antarctica (Figure 1). Many of these widely distributed morphospecies, however, showed no meaningful biogeographic structure.

Clade II includes species of *Corallium* and *Paracorallium* with hemispherical autozooids, an axis with grooves and without polyp rods (*C. elatius* Ridley, *C. kishinouyei* Bayer*, C. konojoi* Kishinouye, *C. niveum* Bayer*, C. secundum* Dana, *Corallium* sp.), others with pits (*Paracorallium nix* Bayer*, P. thrinax* Bayer & Stefani and *Paracorallium* spp.) and characterized by sufficient morphological resolution, reflecting on the phylogenetic pattern of these species, all monophyletic and most with allopatric distributions. For the sympatric species *C. konojoi* and *C. elatius* are restricted to Japan and Taiwan, and are sister species, but *C. secundum*, *C. niveum* and *C. kishinouyei*, endemic to Hawaii, appear in three separate clades (Figure 1).

### Species tree and divergence times

**Figure 2:**
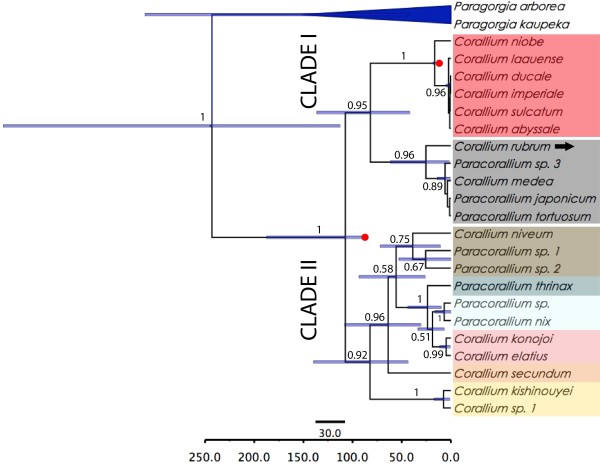
*BEAST species-tree derived from mtDNA. Most probable species tree of Coralliidae from *BEAST. Each node in the tree is labeled with its Bayesian posterior probabilities if it is greater than 0.5. The blue bars represent the 95% HPD interval for the divergence time estimates, which is only available for nodes with posterior probability <0.5. The arrow indicates the position of the type species of the family, *Corallium rubrum.*

Phylogeny of the mtDNA obtained from the calibrated species-tree approach also identified the same main clades, including *Corallium rubrum* within Clade I-A, and a Clade I-B comprising the species with long rod sclerites, cylindrical autozooids and smooth axes (Figure 2). The relationships among coralliid species of Clade II differed slightly in the species-tree when compared with the concatenated gene-tree (Figure 1). Diversification time of Clade I-B was more recent in the species tree (18.0-15.9 million years ago-mya) than in the gene tree (35.2-15.9 mya). In contrast, the diversification times for Clade-II were more ancient in the species tree (136.4-41.7 mya) than in the gene tree (66.3-16.9 mya).

### Species delimitation

**Figure 3:**
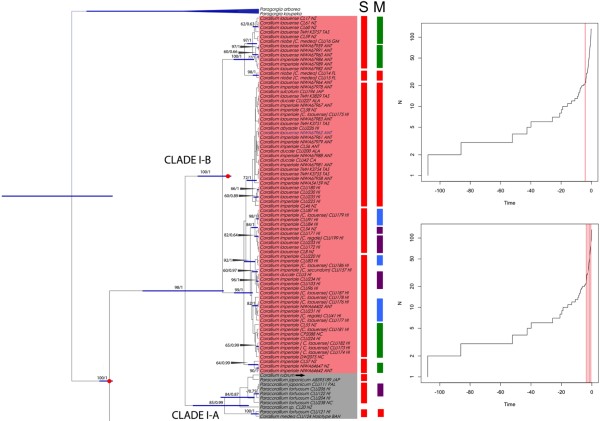
**GMYC model for the mtDNA data for Clade I. **Phylogenetic tree (left), single (S) threshold likelihood solutions to the GMYC model (center), and lineage-through-time plot (right). Log number of lineages (N) vs. time (mya) graph from GMYC analysis based on a Bayesian tree. The threshold between intra- vs. inter-species variation is indicated by a vertical red line. The single threshold GMYC result is indicated for a single line (4.13 mya: red bars). The multiple-threshold GMYC result (lower plot) reflects the results of four thresholds (3.71 mya: red bars, 2.56 mya: green bars, 1.48 mya: blue bars, and 0.68 mya: purple bars).

**Figure 4:**
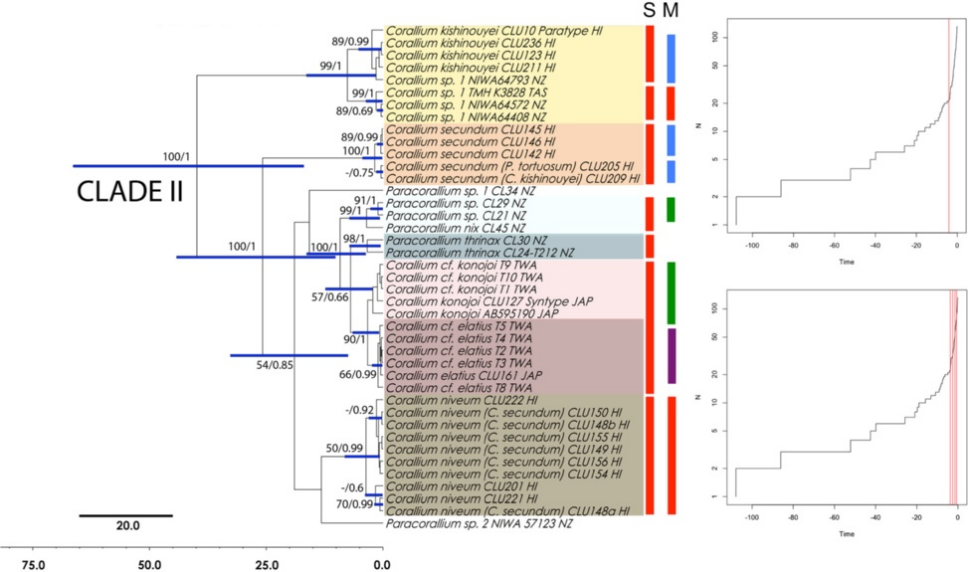
**GMYC model for the mtDNA data for Clade II.** Phylogenetic tree (left), single (S) and multiple (M) threshold likelihood solutions to the GMYC model (center), and lineage-through-time plots (right). Log number of lineages (N) vs. time (mya) graph from GMYC analysis based on a Bayesian tree. The threshold between intra- vs. inter-species variation is indicated by vertical red lines. The single threshold GMYC result (upper plot) is indicated for a single line (4.13 mya: red bars). The multiple-threshold GMYC result (lower plot) reflects the results of four thresholds (3.71 mya: red bars, 2.56 mya: green bars, 1.48 mya: blue bars, and 0.68 mya: purple bars).

A traditional taxonomic study of Clade II delineated 11 morphospecies that were congruent with the general mixed Yule-coalescent (GMYC) model (Figure 3), but resolution within Clade I was lacking (Figure 4). The single-threshold model yielded a total of 16 ML clusters (=GMYC species), including Clades I and II, and the time at which the speciation-coalescent transition occurred was ca. 4.13 mya. The seven GMYC species for Clade II are congruent with the morphological diagnosis for *C. kishinouyei*, *Corallium* sp. 1, *C. secundum*, *C. niveum* and *P. thrinax*, and two cases of merging for *C. konojoi*−*C. elatius* and *Paracorallium* sp.−*P. nix* (Figure 3).

The multiple threshold model yielded more ML clusters (22 GMYC species), and the multiple times at which the speciation-coalescent transition occurred was 3.71, 2.56, 1.48, and 0.68 mya (Figures 3, 4). The eight GMYC species for Clade II, delineated from the multiple-threshold model, were less congruent with the morphological diagnosis in general. In Clade I, the resolution of the species was incomplete and the GMYC delimitation was highly incongruent (Figure 4).

## Discussion

Octocoral species are often described on the basis of diagnostic sclerites and external morphology, but these characters may show phenotypic plasticity, varying within colonies, among habitats or along the life cycle of the colony [43]. Phylogenetic studies in octocorals have showed a lack of resolution using mitochondrial markers, with COI and MSH1 barcodes identifying ca. 70% of morphospecies correctly [44]. Our study presents the most comprehensive phylogeny for the precious corals to date. The study encompasses the geographical range of the family and the majority of recognized species (17 species plus five additional morphospecies; Additional file 1) and includes multiple individuals for 14 of these species from their known distribution ranges. Analyses of these data recover several well-resolved clades in Coralliidae, and many terminal relationships are supported in Clade II. The use of up to five mitochondrial regions showed discordant results in certain parts of the tree, especially for the species in Clade I. The two approaches (concatenated gene-tree vs. species-tree) produced incongruent topologies, but both hypotheses reject the taxonomic status of the coralliid genera, showing that they are not reciprocally monophyletic.

Coralliidae is closely related to Paragorgiidae according to both nuclear and mitochondrial sequences [33], their sclerites are clearly homologous, and a third monotypic family (Sibogagorgiidae) was erected to accommodate a single species [17,45]. *Paracorallium*, according to mitochondrial data, is not a valid taxon. Uda et al. [35] justified the validity of the two genera of Coralliidae based on the genetic distance between *P. japonicum* and *C. konojoi* and differences in their mitochondrial gene arrangements, but our study shows that those differences are meaningless when the genera are densely sampled. Furthermore, they could not include the type species of each genus, *Corallium rubrum* (for the genus *Corallium*) and *C. tortuosum* (for *Paracorallium*). Furthermore, no species from Clade I-B were included, and divergence and anagenesis are no longer used to justify higher taxonomic ranks, in the absence of monophyly. Our results, including the type species for both genera, clearly show that the type species of *Paracorallium* is closer to *C. rubrum* than to some other *Paracorallium* species. Thus we synonymize *Paracorallium***new synonymy** with *Corallium*.

The *Corallium laauense*−*imperiale* clade (I-B) has wide geographical distribution throughout the Pacific Ocean and probably high dispersal abilities. Their colonies branch mainly in one plane, and are irregularly pinnate, with short terminal branchlets arising vertically from the principal branches, and forming arcades or galleries often inhabited by polychaete annelids [21]. The autozooids form cylindrical calices with distinct longitudinal grooves and the coenenchyme sclerites are characterized by 8-radiates in the type material of both species but some differences exist in the presence of double-clubs and the rind and axis coloration [21]. Gray (1867) proposed the use of three genera based on the form of verrucae (autozooid) and the branching pattern: *Corallium*, for *C. rubrum*; *Pleurocorallium* Moroff, for *C. secundum* and *Hemicorallium* Gray for *C. johnsoni*[46]. In the hypothesis here presented Clade I-B included species related to *C. johnsoni * and grouped by Bayer (1956) in a uniform group characterized by a “long spindles in the autozooid verrucae = rods” (*C. johnsoni*, *C. abyssale*, *C. laauense*, *C. imperiale*, *C. niobe*, *C. sulcatum*, *C. ducale*, *C. halmaheirense*, *C. tricolor*, *C. maderense*) [21]. For these reasons we resurrect the genus *Hemicorallium* for Clade I-B characterized by presence of rods and cylindrical autozooids.

In general, the species of Clade I-B (*C. abyssale*, *C. laauense*, *C. imperiale*, *C. niobe*, *C. sulcatum*, *C. ducale*) are very similar in morphology and have been separated by little differences in the calyx’s morphology, the absence/presence of the radiate-sclerites or double-clubs, and coloration. The observed low resolution in mitochondrial loci for these species could be the result of limited genetic variation induced by gene flow, recent diversification times (18.0-15.9 mya or 35.2-15.9 mya depending of the approach), incomplete lineage sorting, or hybridization, among other factors, but could also be explained by phenotypic plasticity in a broadly distributed species complex [43].

In contrast, the species of Clade II exhibit older diversification times (136.4-41.7 mya or 66.3-16.9 mya depending of the approach), more restricted geographical distributions, and limited dispersal abilities, implying a strong role for geographic isolation. In addition, the establishment of discrete and monophyletic clusters is a general consequence of sufficient time for speciation [10]. A similar case of discordance between sister clades has been reported for terrestrial earthworms in the genus *Hormogaster*[47,48]*.* A testable explanation is that the mode and tempo of morphological diversification significantly increased in Clade I as a product of larger effective population sizes, wider geographical and ecological distributions, and, as a consequence, higher selective pressures acting on a morphologic continuum [49]. Such process can be expedited either if the ancestral population of Clade I had larger phenotypic plasticity or if Clades I and II have dramatically different reproductive strategies (e.g., brooders vs. broadcast spawners). Testing either scenario awaits significantly increased, and precisely designed, new deep-sea sampling.

Diversification times estimated with concatenated gene-tree vs. species tree approaches showed significant overestimation within the former approach. This may be common especially for groups with recent divergence events [50], which is consistent with the general pattern of recent divergence times seen in other species trees [26]. Moreover, species delimitation of precious corals should rely on diverse sets of data, including morphological characters and proxies of reproductive isolation (e.g., genetic divergence and reproductive strategy) hopefully in association with biogeographic patterns. Analysis of genomic data and population genetics using fast-evolving markers such as simple sequence repeats (SSRs) and single nucleotide polymorphisms (SNPs) [6,7,12,51] should provide additional valuable information for delimiting recently diverged species.

## Conclusions

The phylogenetic hypotheses recovered by mtDNA concatenated gene trees and species trees reject the taxonomic status of both currently recognized coralliid genera, but it is compatible with the genus *Hemicorallium*, as proposed by Gray (1867), as sister to *Corallium*. *Paracorallium* is polyphyletic and nested within *Corallium*, and thus it is here synonymized with *Corallium*, the type genus of the family. We further re-erect the genus *Hemicorallium*, to include the species (*C. johnsoni*, *C. abyssale*, *C. laauense*, *C. imperiale*, *C. niobe*, *C. sulcatum*, *C. ducale*, *C. halmaheirense*, *C. tricolor*, *C. maderense*). In addition, species delimitation in Clade I-B (species with long rod sclerites, cylindrical autozooids and smooth axes) remains unclear. Molecular resolution of species within Coralliidae differs among Clades I and II. Dated phylogenies indicated that species from Clade I have diverged recently while maintaining gene flow, hybridization or incomplete lineage sorting. This approach is also useful to understand the poor resolution of some species with broad distributions and recent diversification times (Clade I-B) vs. species with allopatric distributions and older diversification times (Clade II) that showed greater levels of mtDNA resolution and clustering.

## Methods

### Tissue collection and DNA extraction

Coralliidae specimens were primarily obtained at the National Museum of Natural History, Smithsonian Institution, Washington D.C., which hosts the most complete collection with approximately 272 lots. Additional material from NIWA Invertebrate Collection (National Institute of Water & Atmospheric Research) and Te Papa Tongarewa, Wellington, New Zealand [7], and recently collected samples from institutions from different areas such as CSIRO (Tasmania) and Sinica Academy (Taiwan) were included [11]. Octocoral specimens were identified through examination of colonial features under a dissecting microscope and dissection of sclerite layers, with observations with both a compound microscope and scanning electron microscopy (SEM) (unpublished data). Genomic DNA extraction was performed with an automated DNA isolation system (AutoGenprep 965, AutoGen Inc.) using the protocol with phenol/chloroform phases and precipitation with ethanol according to modifications made by Herrera et al. [33], or with the DNeasy^®^ Tissue Kit (QIAGEN Inc., Valencia, CA, USA) for some tissue samples (4–5 polyps).

### DNA sequencing

For gene amplification, primers designed for COI, 16S rRNA, 16S rRNA-ND2, and ND3-ND6 sequence regions were used [33,52]. The amplified products were purified using the Exonuclease-I/Shrimp Alkaline Phosphatase (ExoSAP-ITTM, USB Corp.) method. Purified PCR products were sequenced with an automated ABI 3730 XL capillary sequencer using a BigDye Terminator v3.1 kit (Applied Biosystems, Foster City, CA) and purification was done with Sephadex G-50 (Sigma- Aldrich Corp.). Complementary chromatograms were assembled and edited using Geneious Pro v.4.8.5 (Biomatters Ltd., NZ). Nucleotide sequences were aligned for each gene using MUSCLE [53], via the CIPRES portal [54]. For “problematic samples” (i.e. specimens not grouping as expected in the molecular tree), sequencing was performed in three labs (Biommar Lab-UniAndes, Giribet Lab-Harvard University and Museum Support Center-Smithsonian Institution) using different extraction protocols (QIAGEN and automated) and cross-contamination was discarded.

One hundred and twenty-six specimens were sequenced for a fragment of COI, 16S rRNA, and 16S rRNA-ND2. Sequences of the ND3-ND6 fragment were incomplete for six specimens (see Additional file 1). Sequences for *Corallium rubrum* (only 16S rRNA and COI were available), *C. konojoi* (AB595190), *Paracorallium japonicum* (AB595189) and the outgroup *Paragorgia arborea* L. and *P. kaupeka* Sánchez were retrieved from GenBank. Aligned mtDNA sequences were 519 bp for COI, 355 bp for 16S rRNA, 763 bp for 16S rRNA-ND2, and 626 bp for ND3-ND6. All sequences have been deposited in GenBank [11].

### Phylogenetic analysis

#### Concatenated analysis

Tree reconstructions were conducted using Maximum Likelihood and Bayesian inference. Best-fit models of nucleotide substitution were selected for each of the five genes using JModeltest [55] under the Akaike information Criterion (AIC) (COI: HKY+Gamma+I, 16S: GTR+ gamma, ND2 and ND3-ND6: HKY+I). Bayesian analysis was performed using the software package Bayesian Evolutionary Analysis by Sampling Trees (BEAST) version 1.7.4 [56,57]. Divergence dates were estimated from the mtDNA data concatenated to compare with species-tree based estimates using *BEAST part of the BEAST version 1.7.4 and assuming a partitioned scheme employing a relaxed uncorrelated lognormal clock and the Birth-Death process with a uniform prior on birthdate rate as tree prior [56]. Parameters and trees were estimated from two independent runs with four partitions (COI, 16S, ND2, ND3-ND6) using four Markov chains (MCMC) with a chain lengths of 50 millions of generations and default heating values. Trees and parameters were sampled every 1000 generations and the first 10% of the samples were discarded as burn-in. Summary information about sample trees was produced using LogCombiner and TreeAnnotator version 1.7.4. To check the performance of BEAST output for an adequate convergence and mixing quality of all parameters was used Tracer version 1.5 [58] by examining of the loglikelihood values across generation number. In addition, Tracer was used to confirm that post-burn-in trees yielded an effective sample size (ESS) of <200 for all parameters. A fossil of *Corallium* dated to 85.3-84.3 mya based on the occurrence in the Campanian-Maastrichtian of Ukraine [59] and the oldest coralliid fossil known so far was used as the calibration point at the Coralliidae crown. A LogNormal distribution with 95% confidence interval covering this constraint (84.3-111.1 mya) was used for the calibration prior, using offset: 84.3, standard deviation in log space: 2.0 and mean in log space: 0. A second calibration point was included in the Clade I-B, using fossils from the Lower Miocene (Burdigalian) of the Turin Hills (northern Italy) and Capo Milazzo, Sicily, southern Italy (Pleistocene) with typical tunnels most probably produced by commensal worms like the species in this Clade (e.g. *C.imperiale*) [60]. We used the oldest fossil known from lower Miocene to calibrate the Clade I-B (20.4-15.9 mya). A LogNormal distribution with 95% confidence interval covering this constraint (42.7-15.9 mya) was used for the calibration prior, using next values for the offset: 15.9, standard deviation in log space: 2.0 and mean in log space: 0. We understand the limitations and uncertainties of dating with a few calibration points [61,62]. Moreover, we used this calibration to know the relative divergence among clades before that absolute time.

ML trees were estimated by means RAxML 7.3.2 using GTR nucleotide-substitution model + gamma distribution and GTRCAT model to be used during the inference of the best tree and bootstrapping phase respectively [63,64] via the CIPRES portal [54]. Bootstrap support for the mtDNA-concatenated tree was assessed with 1000 replicates that were specified automatically with the Majority Rule criterion.

#### Species tree and divergence times

Species-tree analyses and divergence time estimates from a multilocus (four partition of mitochondrial genes) and multi-individual (< 2 individuals per species) data set followed the *BEAST approach [27] in BEAST. Although species-tree analyses are usually performed using multilocus data, inference from a single locus is possible (see details in [26,27]), and the motivation to use several mitochondrial regions as independent markers is that it can bring some measure of rate heterogeneity that might help to resolve the phylogenetic trees. Additionally is preferable the species tree approach for divergence time estimates because they take into account the gene divergence within the ancestral population, preventing overestimations in their calculations [65].

In the same way as the gene-tree concatenated analysis from above, all individuals were used (128 individuals representing 17 species of Coralliidae), and the same MCMC options, burn-in and calibration point were applied to the species tree. The default options for species tree priors (Yule process) and population size models (piecewise linear and constant root) were used in this analysis.

#### Species delimitation

Species limits were established using a generalized mixed Yule coalescent (GMYC) model [8,66]. In brief the GMYC model attempts to identify the species delimitation analyzing the shift in branching rates on a tree that contains multiple samples per species. The GMYC detects the predicted difference in branching rate under two modes of lineage evolution (within and among species), evaluating the point of highest likelihood of the transition [8]. The *gmyc* function using the R statistical language and the APE library in conjunction with the package "gmyc.pkg.0.9.6.R" was used on the ultrametric concatenated gene tree resulting from the Bayesian analyses to do a single-threshold [8,66] and a multiple-threshold [5] method.

## Competing interests

The authors declare that they have no competing interests.

## Authors’ contributions

NEA participated in the design of the study and coordination, secured funding, carried out the molecular sequencing, the phylogenetic analysis and drafted the manuscript. GG obtained samples of *C. rubrum*, additional sequences of Coralliidae and helped to critically revise the manuscript. JAS conceived the study, obtained samples of Coralliidae, participated in its design and coordination and helped to write the manuscript. The authors read and approved the final manuscript.

## Supplementary Material

Additional file 1 **Sequences and specimens of coralliids used in this study.** Acronyms as follows: National Museum of Natural History of the Smithsonian Institution, USA (USNM); The National Institute of Water and Atmospheric Research, New Zealand (NIWA); CSIRO Tasmania (TMH); Academia Sinica, Taiwan (ASIZ). Sequences not generated in this study are in bold. Source: ^1^Herrera et al. 2009, ^2^Uda et al. 2011, ^3^Calderon et al. 2006, ^4^Tinti et al. (unpublished), ^5^France S.C. & Thoma (unpublished). Click here for file

## References

[B1] de Queiroz K Species concepts and species delimitation Syst Biol 2007 56 6 879 886 10.1080/10635150701701083 18027281

[B2] Carstens B Dewey T Species delimitation using a combined coalescent and information-theoretic approach: an example from North American Myotis bats Syst Biol 2010 59 4 400 414 10.1093/sysbio/syq024 20547777 PMC2885268

[B3] Knowles L Carstens B Delimiting species without monophyletic gene trees Syst Biol 2007 56 6 887 895 10.1080/10635150701701091 18027282

[B4] Lim GS Balke M Meier R Determining species boundaries in a world full of rarity: singletons, species delimitation methods Syst Biol 2011 60 165 169 21482553 10.1093/sysbio/syr030

[B5] Monaghan M Wild R Elliot M Fujisawa T Balke M Inward D Lees D Ranaivosolo R Eggleton P Barraclough T Accelerated species inventory on Madagascar using coalescent-based models of species delineation Syst Biol 2009 58 298 311 10.1093/sysbio/syp027 20525585

[B6] Noble D Qi Y Fu J Species delineation using Bayesian model-based assignment tests: a case study using Chinese toad-headed agamas (genus Phrynocephalus) BMC Evol Biol 2010 10 1 197 10.1186/1471-2148-10-197 20579368 PMC2904330

[B7] Pinzón J LaJeunesse T Species delimitation of common reef corals in the genus Pocillopora using nucleotide sequence phylogenies, population genetics and symbiosis ecology Mol Ecol 2011 20 311 325 10.1111/j.1365-294X.2010.04939.x 21091563

[B8] Pons J Barraclough T Gomez-Zurita J Cardoso A Duran D Hazell S Kamoun S Sumlin W Vogler A Sequence-based species delimitation for the DNA taxonomy of undescribed insects Syst Biol 2006 55 4 595 609 10.1080/10635150600852011 16967577

[B9] Yang Z Rannala B Bayesian species delimitation using multilocus sequence data Proc Natl Acad Sci 2010 107 9264 9269 10.1073/pnas.0913022107 20439743 PMC2889046

[B10] Papadopoulou A Bergsten J Fujisawa T Monaghan M Barraclough T Vogler A Speciation and DNA barcodes: testing the effects of dispersal on the formation of discrete sequence clusters Philosophical Transactions of the Royal Society B 2008 363 2987 2996 10.1098/rstb.2008.0066 PMC2607311 18522916

[B11] Sites J Jr Marshall J Operational criteria for delimiting species Annu Rev Ecol Evol Syst 2004 35 199 227 10.1146/annurev.ecolsys.35.112202.130128

[B12] Hausdorf B Hennig C Species delimitation using dominant and codominant multilocus markers Syst Biol 2010 59 491 503 10.1093/sysbio/syq039 20693311

[B13] Sites J Jr Marshall J Delimiting species: a Renaissance issue in systematic biology Trends Ecol Evol 2003 18 9 462 470 10.1016/S0169-5347(03)00184-8

[B14] Wiens JJ Penkrot TA Delimiting species using DNA and morphological variation and discordant species limits in spiny lizards (Sceloporus) Syst Biol 2002 51 1 69 91 10.1080/106351502753475880 11943093

[B15] Wiens J Species delimitation: new approaches for discovering diversity Syst Biol 2007 56 6 875 878 10.1080/10635150701748506 18027280

[B16] Bayer F Key to the genera of Octocorallia exclusive of Pennatulacea (Coelenterata: Anthozoa), with diagnoses of new taxa Proceedings of the Biological Society of Washington 1981 94 3 902 947

[B17] Sánchez JA Systematics of the bubblegum corals (Cnidaria: Octocorallia: Paragorgiidae) with description of new species from New Zealand and the Eastern Pacific Zootaxa 2005 1014 1 72

[B18] Sánchez J A new genus of Atlantic octocorals (Octocorallia: Gorgoniidae): systematics of gorgoniids with asymmetric sclerites J Nat Hist 2007 41 493 509 10.1080/00222930701237315

[B19] Sánchez J Systematics of the candelabrum gorgonian corals (Eunicea Lamouroux; Plexauridae; Octocorallia; Cnidaria) Zoological J Linnean Soc 2009 157 2 237 263 10.1111/j.1096-3642.2008.00515.x

[B20] Sánchez J Wirshing H A field key to the identification of tropical western Atlantic zooxanthellate octocorals (Octocorallia: Cnidaria) Caribbean J Sci 2005 41 3 508 522

[B21] Bayer F Descriptions and redescriptions of the Hawaiian octocorals collected by the US Fish Commission steamer "Albatross" (2. Gorgonacea: Scleraxonia) Pacific Science 1956 10 1 67 95

[B22] Cummings M Neel M Shaw K A genealogical approach to quantifying lineage divergence Evolution 2008 62 9 2411 2422 10.1111/j.1558-5646.2008.00442.x 18564377

[B23] O'Meara B New heuristic methods for joint species delimitation and species tree inference Syst Biol 2010 59 1 59 73 10.1093/sysbio/syp077 20525620 PMC5841455

[B24] Rosenberg N Nordborg M Genealogical trees, coalescent theory and the analysis of genetic polymorphisms Nat Rev Genet 2002 3 5 380 390 10.1038/nrg795 11988763

[B25] Degnan J Rosenberg N Gene tree discordance, phylogenetic inference and the multispecies coalescent Trends Ecol Evol 2009 24 6 332 340 10.1016/j.tree.2009.01.009 19307040

[B26] McCormack J Heled J Delaney K Peterson A Knowles L Calibrating divergence times on species trees versus gene trees: implications for speciation history of Aphelocoma jays Evolution 2011 65 1 184 202 10.1111/j.1558-5646.2010.01097.x 20681982

[B27] Heled J Drummond A Bayesian inference of species trees from multilocus data Mol Biol Evol 2010 27 3 570 580 10.1093/molbev/msp274 19906793 PMC2822290

[B28] Bayer F Three new species of precious coral (Anthozoa: Gorgonacea, genus Corallium) from Pacific waters Proceedings of the Biological Society of Washington 1996 109 2 205 228

[B29] Bayer F Cairns S A new genus of the scleraxonian family Coralliidae (Octocorallia: Gorgonacea) Proceedings of the Biological Society of Washington 2003 116 1 222 228

[B30] Bayer F Contributions to the nomenclature, systematics, and morphology of the Octocorallia Proceedings of the United States National Museum 1955 105 207 220 10.5479/si.00963801.105-3357.207

[B31] Bayer F The genus Corallium (Gorgonacea: Scleraxonia) in the western North Atlantic Ocean B Mar Sci 1964 14 3 465 478

[B32] Bayer F Grasshoff M Verseveldt J Illustrated trilingual glossary of morphological and anatomical terms applied to Octocorallia 1983 Leiden: E.J. Brill

[B33] Herrera S Baco A Sánchez J Molecular systematics of the bubblegum coral genera (Paragorgiidae, Octocorallia) and description of a new deep-sea species Mol Phylogenet Evol 2009 55 123 135 20025983 10.1016/j.ympev.2009.12.007

[B34] Sánchez J McFadden C France S Lasker H Molecular phylogenetic analyses of shallow-water Caribbean octocorals Mar Biol 2003 142 5 975 987

[B35] Uda K Komeda Y Koyama H Koga K Fujita T Iwasaki N Suzuki T Complete mitochondrial genomes of two Japanese precious corals, Paracorallium japonicum and Corallium konojoi (Cnidaria, Octocorallia, Coralliidae): notable differences in gene arrangement Gene 2011 476 27 37 10.1016/j.gene.2011.01.019 21310221

[B36] McFadden C France S Sánchez J Alderslade P A molecular phylogenetic analysis of the Octocorallia (Cnidaria: Anthozoa) based on mitochondrial protein-coding sequences Mol Phylogenet Evol 2006 41 3 513 527 10.1016/j.ympev.2006.06.010 16876445

[B37] Bramanti L Magagnini G Demaio L Santangelo G Recruitment, early survival and growth of the Mediterranean red coral (L 1758), a 4-year study J Exp Mar Biol Ecol 2005 314 1 69 78 10.1016/j.jembe.2004.08.029

[B38] Roark E Guilderson T Dunbar R Ingram B Radiocarbon-based ages and growth rates of Hawaiian deep-sea corals Mar Ecol Prog Ser 2006 327 1 14

[B39] Grigg R Resource management of precious corals Marine Ecology 1984 5 1 57 74 10.1111/j.1439-0485.1984.tb00307.x

[B40] Grigg R Precious coral fisheries of Hawaii and the US Pacific Islands Marine Fisheries Review 1993 55 2 50 60

[B41] Grigg R Precious corals in Hawaii: discovery of a new bed and revised management measures for existing beds Marine Fisheries Review 2002 64 1 13 20

[B42] Tsounis G Rossi S Grigg R Santangelo G Bramanti L Gili J The exploitation and conservation of precious corals Oceanography and Marine Biology: An Annual Review 2010 48 161 212

[B43] Sanchez J Aguilar C Dorado D Manrique N Phenotypic plasticity and morphological integration in a marine modular invertebrate BMC Evol Biol 2007 7 122 10.1186/1471-2148-7-122 17650324 PMC1959521

[B44] McFadden C Benayahu Y Pante E Thoma J Nevarez P France S Limitations of mitochondrial gene barcoding in Octocorallia Mol Ecol Resour 2011 11 1 19 31 10.1111/j.1755-0998.2010.02875.x 21429097

[B45] Verseveldt J Further studies on Octocorallia Zoologische Mededelingen 1942 24 159 186

[B46] Gray J Additional note on Corallium johnsoni Proceedings of the Zoological Society of London 1867 35 125 127

[B47] Novo M Almodóvar A Fernández R Trigo D Díaz-Cosín D Giribet G Appearances can be deceptive: different diversification patterns within a group of Mediterranean earthworms (Oligochaeta, Hormogastridae) Mol Ecolin press 10.1111/j.1365-294X.2012.05648.x 22805584

[B48] Novo M Almodóvar A Fernández R Trigo D Díaz-Cosín D Cryptic speciation of hormogastrid earthworms revealed by mitochondrial and nuclear data Mol Phylogenet Evol 2010 56 507 512 10.1016/j.ympev.2010.04.010 20398776

[B49] Jablonski D Species selection: theory and data Annu Rev Ecol Evol Syst 2008 39 1 501 524 10.1146/annurev.ecolsys.39.110707.173510

[B50] Carstens B Knowles L Estimating species phylogeny from gene-tree probabilities despite incomplete lineage sorting: an example from Melanoplus grasshoppers Syst Biol 2007 56 3 400 411 10.1080/10635150701405560 17520504

[B51] Shaffer H Thomson R Delimiting species in recent radiations Syst Biol 2007 56 6 896 906 10.1080/10635150701772563 18066926

[B52] France S Rosel P Agenbroad J Mullineaux L Kocher T DNA sequence variation of mitochondrial large-subunit rRNA provides support for a two-subclass organization of the Anthozoa (Cnidaria) Mol Mar Biol Biotechnol 1996 5 1 15 28 8869515

[B53] Edgar R MUSCLE: multiple sequence alignment with high accuracy and high throughput Nucleic Acids Res 2004 32 5 1792 1797 10.1093/nar/gkh340 15034147 PMC390337

[B54] Miller M Pfeiffer W Schwartz T Creating the CIPRES Science Gateway for inference of large phylogenetic trees Proceedings of the Gateway Computing Environments Workshop (GCE) 2010 New Orleans 1 8

[B55] Posada D jModelTest: phylogenetic model averaging Mol Biol Evol 2008 25 7 1253 1256 10.1093/molbev/msn083 18397919

[B56] Drummond A Rambaut A BEAST: Bayesian evolutionary analysis by sampling trees BMC Evol Biol 2007 7 1 214 10.1186/1471-2148-7-214 17996036 PMC2247476

[B57] Drummond AJ Suchard MA Xie D Rambaut A Bayesian phylogenetics with BEAUti and the BEAST 1.7 Mol Biol Evol 2012 29 1969 1973 10.1093/molbev/mss075 22367748 PMC3408070

[B58] Rambaut A Drummond A Tracer v1.5 2009 Available from http://tree.bio.ed.ac.uk/software/tracer/

[B59] Schlagintweit F Gawlick H The incertae sedis Carpathoporella Dragastan, 1995, from the Lower Cretaceous of Albania: skeletal elements (sclerites, internodes/branches, holdfasts) of colonial octocorals Facies 2009 55 4 553 573 10.1007/s10347-009-0185-5

[B60] Vertino A Zibrowius H Rocca M Taviani M Bussoletti E, Cottingham D, Bruckner A, Roberts G, Sandulli R Fossil Coralliidae in the Mediterranean basin Proceedings of the international workshop on red coral science, management, and trade: lessons from the mediterranean 2010 Silver Spring: NOAA Technical Memorandum CRCP-13 233

[B61] Sauquet H Ho SYW Gandolfo MA Jordan GJ Wilf P Cantrill DJ Bayly MJ Bromham L Brown GK Carpenter RJ Testing the impact of calibration on molecular divergence times using a fossil-rich group: the case of nothofagus (fagales) Syst Biol 2012 61 2 289 313 10.1093/sysbio/syr116 22201158

[B62] Warnock RCM Yang Z Donoghue PCJ Exploring uncertainty in the calibration of the molecular clock Biol Lett 2012 8 1 156 159 10.1098/rsbl.2011.0710 21865245 PMC3259980

[B63] Stamatakis A RAxML-VI-HPC: maximum likelihood-based phylogenetic analyses with thousands of taxa and mixed models Bioinformatics 2006 22 21 2688 2690 10.1093/bioinformatics/btl446 16928733

[B64] Stamatakis A Hoover P Rougemont J A fast bootstrapping algorithm for the RAxML Web-servers Syst Biol 2008 57 5 758 771 10.1080/10635150802429642 18853362

[B65] Edwards S Beerli P Perspective: gene divergence, population divergence, and the variance in coalescence time in phylogeographic studies Evolution 2000 54 1839 1854 11209764 10.1111/j.0014-3820.2000.tb01231.x

[B66] Fontaneto D Kaya M Herniou E Barraclough T Extreme levels of hidden diversity in microscopic animals (Rotifera) revealed by DNA taxonomy Mol Phylogenet Evol 2009 53 1 182 189 10.1016/j.ympev.2009.04.011 19398026

